# Three-Dimensionally-Printed Polyether-Ether-Ketone Implant with a Cross-Linked Structure and Acid-Etched Microporous Surface Promotes Integration with Soft Tissue

**DOI:** 10.3390/ijms20153811

**Published:** 2019-08-04

**Authors:** Xiaoke Feng, Hao Yu, Huan Liu, Xiaonan Yu, Zhihong Feng, Shizhu Bai, Yimin Zhao

**Affiliations:** State Key Laboratory of Military Stomatology & National Clinical Research Center for Oral Diseases & Shaanxi Key Laboratory of Stomatology, Department of Prosthodontics, School of Stomatology, The Fourth Military Medical University, Xi’an 710032, China

**Keywords:** polyether-ether-ketone, three-dimensional printing, injection molding, acid-etch, soft tissue

## Abstract

Polyether-ether-ketone (peek) is one of the most common materials used for load-bearing orthopedic devices owing to its radiolucency and favorable mechanical properties. However, current smooth-surfaced peek implants can lead to fibrous capsule formation. To overcome this issue, here, peek specimens with well-defined internal cross-linked structures (macropore diameters of 1.0–2.0 mm) were fabricated using a three-dimensional (3D) printer, and an acid-etched microporous surface was achieved using injection-molding technology. The cell adhesion properties of smooth and microporous peek specimens was compared in vitro through a scanning electron microscope (SEM), and the soft tissue responses to the both microporous and cross-linked structure of different groups were determined in vivo using a New Zealand white rabbit model, and examined through histologic staining and separating test. The results showed that the acid-etched microporous surface promoted human skin fibroblasts (HSF) adherence, while internal cross-linked structure improved the ability of the peek specimen to form a mechanical combination with soft tissue, especially with the 1.5 mm porous specimen. The peek specimens with both the internal cross-linked structure and external acid-etched microporous surface could effectively promote the close integration of soft tissue and prevent formation of fibrous capsules, demonstrating the potential for clinical application in surgical repair.

## 1. Introduction

Polyetheretherketone (PEEK) is a synthetic, tooth-colored polymeric material that has been used as a biomaterial in orthopedics for many years [1,2,3]. It is a semicrystalline material having a melting point around 335 °C. PEEK can be modified either by the addition of functionalized monomers (pre-polymerization) or post-polymerization modifications by chemical processes such as sulphonation, amination, and nitration [4]. At present, peek is mainly used in the following aspects: maxillofacial skull reconstruction, femoral reconstruction, and hip joint replacement, spinal surgery, cardiac surgery, and oral implant [5]. In addition, due to their high strength properties, PEEK has been used in multiple fields of orthopedics, including femoral prosthesis transplantation, total hip arthroplasty, and hip surface arthroplasty [5]. The use of PEEK cages has become increasingly popular due to their excellent elasticity, X-ray transmissibility, and MRI compatibility [6].

The long-term success of polyether-ether-ketone (peek) implants relies on not only osseointegration but also achieving stable combination with the soft tissue, which remains a clinical challenge [7,8,9]. Such integration of soft tissue with the surface of a peek implant serves as a necessary barrier to prevent bacterial invasion in the deeper tissues surrounding the implant [7,10,11]. The chemical inertness of peek is the main contributor to its poor ability for combining with soft tissue, which causes gaps between the peek implant and soft tissue [12,13]. Therefore, a rational design of the surface and internal spatial structure of peek implants is essential to enable faster soft tissue combination establishment with long-term maintenance.

In general, a smooth surface is considered to be most suitable for the formation of a stable biological seal with soft tissue [14]. However, a smooth peek surface can lead to the formation of a fibrous capsule, which would prevent the necessary contraction of fibrous connective tissues for wound closure during healing [15]. Several recent reports indicated that a microporous surface of an implant might be more effective for establishing a broad and tight attachment to the soft connective tissue at the early stages of healing [16,17,18]. Physical spraying methods [19] or acid etching [20] are the most common methods used to form a bioactive surface layer with a microporous structure on peek. Waser-Althaus [21] applied the O_2_/Ar or NH4 plasma to treat the PEEK surface. They demonstrated an increased adhesion, proliferation, and osteogenic differentiation of adipose tissue-derived mesenchymal stem cells (adMSC) on plasma-treated PEEK. Brydone et al. [22] fabricated novel nanopatterned PEEK rods, etched these PEEK rods with O_2_ plasma to improve their bioactivity and then implanted them into a femoral defect rabbit model. However, physical spraying techniques only result in limited mechanical integration with soft tissue, and the sprayed layer is only weakly bound to the peek surface and does not offer long-term stability [23]. Moreover, acid etching of a microporous surface also provides poor stability of the bond to soft tissue, which can be easily separated from the peek implant when moved [24]. Therefore, there is still a need for new techniques to enhance the combination of soft tissue and a peek implant.

Three-dimensional (3D) printing technology is now widely applied in the field of tissue engineering and offers promise for the future development of peek materials to expand their clinical applications. Owing to rapid developments in 3D printing technology, it is now possible to produce precise internal secondary structures with well-defined shapes [25,26]. Indeed, in the last few decades, a variety of materials with specifically engineered architectures have been developed by 3D printing like poly lactic-co-glycolic acid (PLGA), polylactic acid (PLA), polyvinyl acetate (PVA), hydroxyapatite (HA) and so on, leading to significant advances in various fields of medicine and biology. Accordingly, we were particularly interested in fabricating precisely designed feature sizes, and shapes inside a peek implant using 3D printing technology. However, existing research suggests that 3D printing technology cannot realize a peek specimen with a precise internal secondary structure, and can only fabricate peek specimens through crowding silk accumulation, resulting in only a complete first-order structure [27]. Moreover, computer-aided design/computer-aid manufacturing (CAD/CAM) technology also could not fabricate peek specimens with a precise internal secondary structure [28]. Therefore, in this study, we combined 3D printing with injection-molding technology to realize a peek implant design with an exact secondary structure that would facilitate stable and long-term integration with soft tissues for improved outcome in orthopedic implants for healing and tissue regeneration.

Accordingly, we used 3D printing with injection-molding technology to precisely design a cross-linked structure inside a peek specimen with and without an acid-etched microporous surface. We then compared the effects of the size of the internal network and the microporous or smooth surface on the ability for mechanical combination with soft tissue in vitro using human skin fibroblasts and in vivo in a New Zealand white rabbit model. These findings can provide a theoretical foundation for the possible clinical application of this peek implant design.

## 2. Results

### 2.1. Surface Characterization

**Figure 1:**
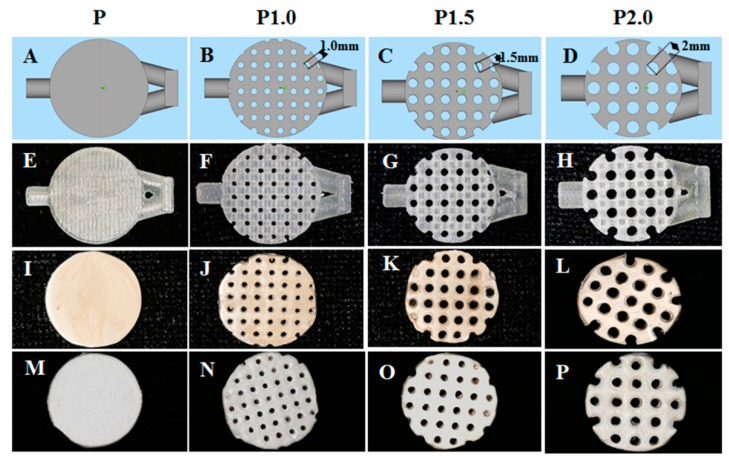
Schematic design and photographs of peek specimens. Computer-designed images of specimens with a cross-linked structure of different sizes: (**A**) P, (**B**) P1.0, (**C**) P1.5, and (**D**) P2.0. Resin molds fabricated by 3D printing of different sizes: (**E**) P, (**F**) P1.0, (**G**) P1.5, and (**H**) P2.0. Peek specimens fabricated by injection-molding technology with different sizes: (**I**) P, (**J**) P1.0, (**K**) P1.5, and (**L**) P2.0. Acid-etched peek specimens fabricated by injection-molding technology with different sizes: (**M**) P, (**N**) P1.0, (**O**) P1.5, and (**P**) P2.0.

Photographs of the peek specimens are shown in Figure 1. The specimens were first designed in the software with a cross-linked structure containing casting and exhaust channels, which showed pores on the surface with a pore diameter of 1.0 mm, 1.5 mm, and 2.0 mm respectively (Figure 1A–D). The resin molds fabricated by 3D printing clearly showed the cross-linked structure inside the specimens after the supporting material was removed (Figure 1E–H). Specimens without a sulfuric acid etch were polished to obtain a mirror-like smooth surface (Figure 1I–L), with the corresponding groups: group P, group P1.0, group P1.5, and group P2.0 and the sulfuric acid-etched specimens (Figure 1M–P) were covered with a much rougher chalky surface, with the corresponding groups: group SP, group SP1.0, group SP1.5, group SP2.0.

**Figure 2:**
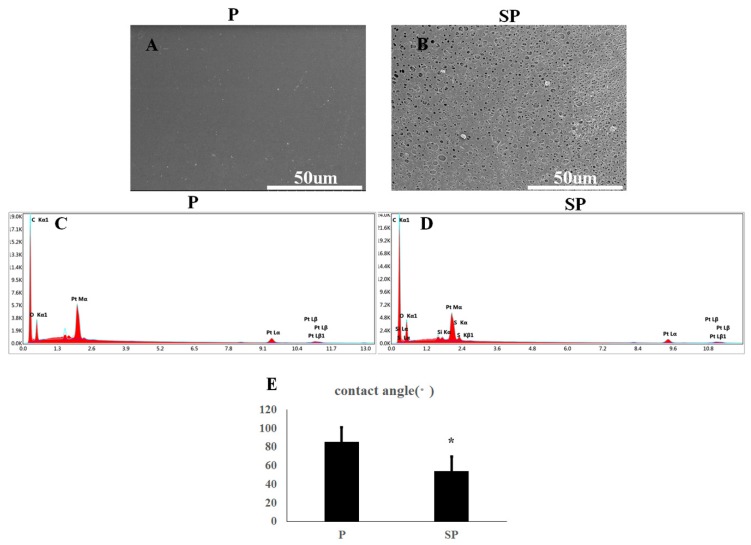
Surface characterization of peek specimens. SEM images of peek specimen (**A**) P and (**B**) SP. Elemental components of peek specimens (**C**) P and (**D**) SP determined by XDS. Water contact angles of peek specimens (**E**) P and (**F**) SP.* *p* < 0.05.

SEM images of the surface of each specimen are shown in Figure 2. Specimen P was smooth with minimally detectable surface features (Figure 2A); however, specimen SP was visually rough with a randomly distributed mesh (Figure 2B). Surface energy spectrum analysis showed no residual S element on the material surface of specimen SP except for the main components of peek, carbon, and oxygen (Figure 2C,D). The water contact angle of the specimen P was significantly higher than that of the specimen subjected to acid corrosion (Figure 2E, *p* < 0.001). 

**Figure 3:**
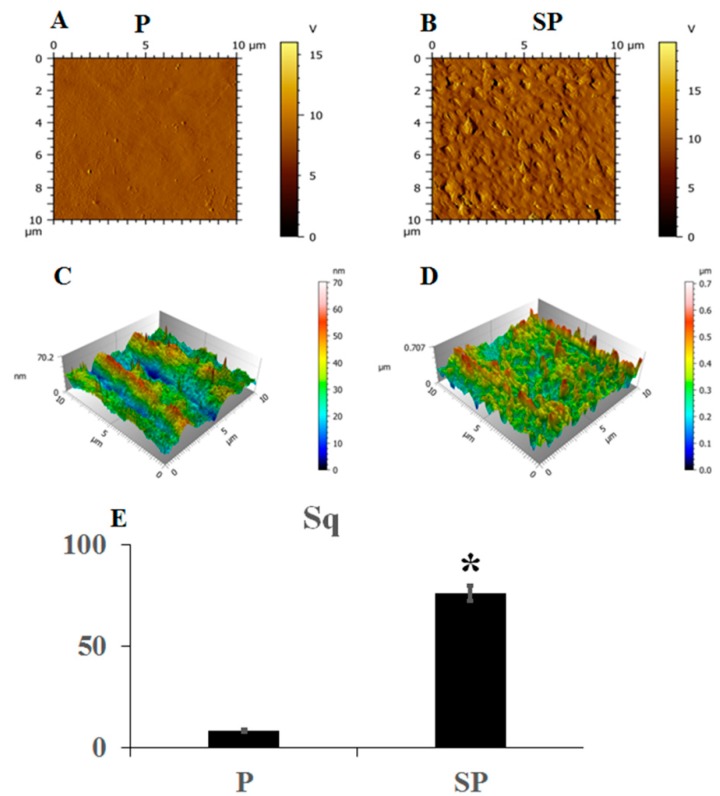
Atomic force microscopy (AFM) images of peek specimens (**A**) P and (**B**) SP. Three-dimensional fluctuating images of peek specimens (**C**) P and (**D**) SP for comparison of the surface roughness. * *p* < 0.05.

Atomic force microscopy (AFM) showed that the surface of peek specimen P (Figure 3A) was smooth without an undulating grain, whereas the surface of specimen SP, which had been acid-etched, was uneven and rough (Figure 3B). Three-dimensional reconstruction of the surface morphology of the two groups of peek specimens showed that the smooth peek specimen showed moderate fluctuations while the acid-etched peek specimen showed significantly greater fluctuations (Figure 3C,D). The surface roughness of specimen P was significantly lower than that of the specimen SP (Figure 3E, *p* < 0.001).

### 2.2. Mechanical Properties of Peek Specimens

**Figure 4:**
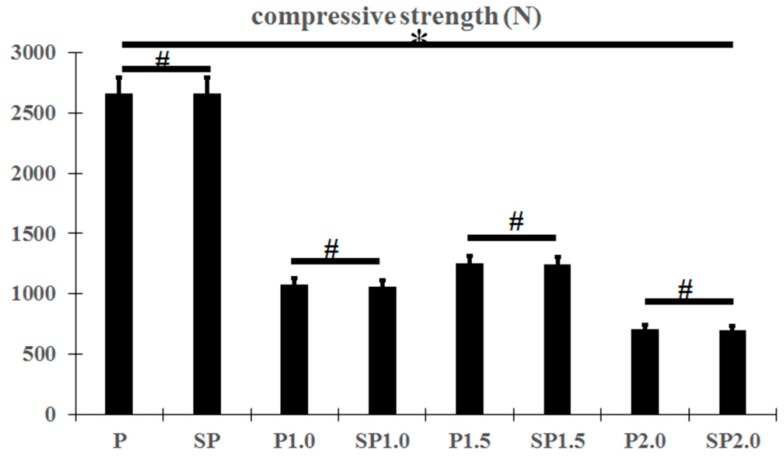
Compressive strength of peek specimens. * *p* < 0.05, # *p* > 0.05.

The compressive strength of the peek specimens decreased with an increase of the pore diameter of the peek, and groups P1.5 and SP1.5 showed much higher compressive strength than the other specimens with a cross-linked structure; however, there was no change in the compressive strength of the samples of the same type before and after acid etching (Figure 4; *p* < 0.05).

### 2.3. Cell Adherence and Soft Tissue Integration of Peek Structures In Vitro

**Figure 5:**
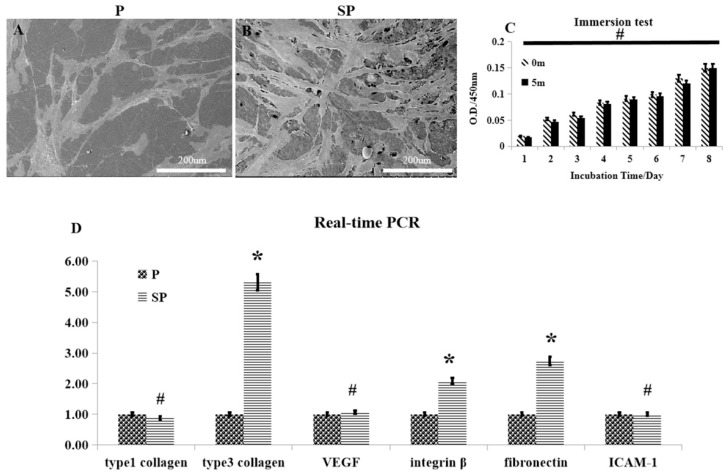
The biological behavior of human skin fibroblasts (HSFs) on the surface of the peek materials. (**A**,**B**) SEM images of HSFs adhering on the peek specimens, and acid-etched peek specimens. (**C**) Comparison of the influence of P and SP for HSF adhesion. (**D**) RT-PCR of collagen I, collagen III, VEGF, integrin-β, ICAM-1, and GAPDH 5 days after spreading the HSFs on the specimens. * *p* < 0.05, # *p* > 0.05.

There was a larger number of HSFs seeded on the surface of peek specimens after acid etching than on those without acid etching (Figure 5A,B). However, the proliferation of cells after culture in the leach liquor of each peek specimen was not significantly affected by acid etching (Figure 5C; *p* > 0.05). RT-PCR further showed no difference in the mRNA expression levels of type I collagen, VEGF, and ICAM-1, whereas the mRNA expression levels of type III collagen, integrin-β, and fibronectin were significantly higher in peeks of the SP groups, which are all closely linked with the growth of soft tissues.

### 2.4. Effect of Peak Structure and Surface on Soft Tissue Combination In Vivo

**Figure 6:**
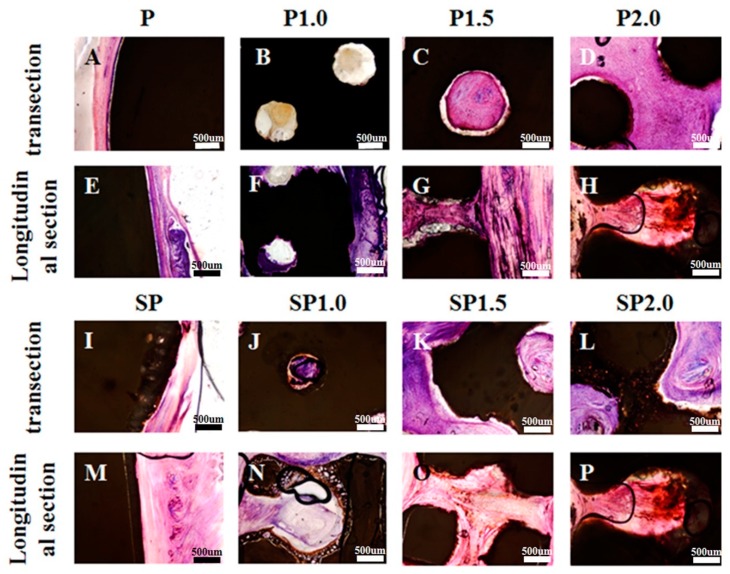
Hematoxylin and eosin (HE) staining images of different transplanted peek specimens after 4 weeks. (**A**) Transection HE staining images of group (**A**) P, (**B**) P1.0, (**C**) P1.5, (**D**) P2.0, (**I**) SP, (**J**) SP1.0, (**K**) SP1.5, and (**L**) SP2.0 with soft tissue. Longitudinal sections of HE staining images of group (**E**) P, (**F**) P1.0, (**G**) P1.5, and (**H**) P2.0, (**M**) SP, (**N**) SP1.0, (**O**) SP1.5, and (**P**) SP2.0 with soft tissue.

At 4 weeks after subcutaneous transplantation of peek specimens in New Zealand white rabbits, HE staining showed that the P group had formed interspaces between the soft tissue and peek specimen in both transection and longitudinal section views (Figure 6A,E). No such interspace was observed in the SP group, and some soft tissue could be seen growing into the microporous acid-etched surface, which enhanced the connection between the soft tissue and peek (Figure 6I,M). The P1.0, P1.5, P2.0, SP1.0, SP1.5, and SP2.0 groups all had soft tissues growing inside the peek in both transectional and longitudinal views. Some interspaces between the soft tissue and peek specimens were observed in groups P1.0, P1.5, and P2.0 (Figure 6B–D) but not in groups SP1.0, SP1.5, and SP2.0 (Figure 6J–L) in transectional view. Moreover, the SP1.0, SP1.5, and SP2.0 groups had soft tissues growing on the microporous acid-etched surface. However, the HE staining also showed that soft tissues partially grew into the P1.0 and SP1.0 specimens, while greater connection was observed in the P1.5, P2.0, SP1.5, and SP2.0 groups.

**Figure 7:**
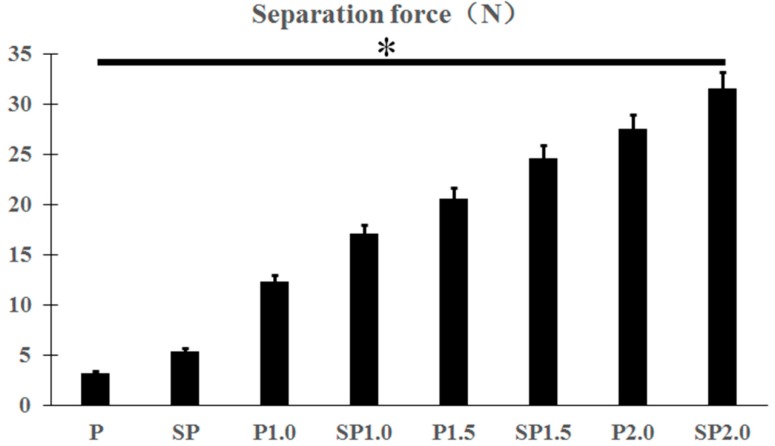
Separation force of different peek specimens with soft tissue. * *p* < 0.05.

Indeed, at 4 weeks after transplantation, the specimens with a cross-linked structure clearly showed a higher binding force with the soft tissue than that of specimens without a cross-linked structure (Figure 7; *p* < 0.05). The force required for soft tissue separation in each experimental group was P (3.19 ± 0.69), SP (5.43 ± 0.37), P1.0 (12.35 ± 1.54), SP1.0 (17.09 ± 1.28), P1.5 (20.59 ± 0.85), SP1.5 (24.57 ± 1.25), P2.0 (27.47 ± 0.38), and SP2.0 (31.56 ± 0.81). The adhesion force between the soft tissue and peek increased with the increase in the diameter of the cross-linked structure. There was also a significant difference in the binding force between the acid-etched and smooth specimens harboring the same cross-linked structure when separated with soft tissues. Similarly, the binding force between specimens and the soft tissue also increased with the increase of the diameter of the cross-linked structure among only acid-etched specimens.

## 3. Discussion

The main challenge of peek implants in clinical application as orthopedic devices is the formation of a fibrous capsule, which is mainly due to improper closure of the implant-soft tissue interface caused by the inert property of peek [29]. Thus, optimal performance of peek implants requires achieving rapid mechanical combination with the soft tissue and long-term stability. Various factors affect the combination between a peek implant and soft tissue, including the surgical techniques, implant design, and implant surface morphology [30]. Most of the currently used peek implants have a rough surface to promote the adhesion of cells and surrounding tissue [11,31]. This rough surface is generally achieved by physically spraying bioactive substances onto peek [21,22,32]; however, the sprayed layer is only weakly bound to the peek surface, which may desquamate when making contact with the surrounding soft tissue over time [33]. A smooth surface can lead to the formation of a fibrous capsule and an unsatisfactory combination with the tissue, resulting in ultimate transplant failure [34]. To overcome these limitations, there have been many attempts to improve the bioactivity of smooth peek surfaces using antibacterial coatings [35]; however, few studies have concentrated on methods for enhancing the mechanical combination of a peek implant and soft tissue.

As an alternative to physical spraying, acid etching can be used to fabricate a peek implant with a rough surface, which has also been shown to improve the bioactivity of the implant [36]. Moreover, previous studies indicated that a suitably micro-structured peek implant surface could potentially achieve a more rigid mechanical combination with soft tissue [16,17]. Indeed, in the present study, the peek with a microporous coating fabricated with acid etching promoted the initial adhesion, viability, spread, and fibronectin secretion of HSF cells. Initial cell adhesion was considered to be the pivotal step for subsequent cell-biomaterial interactions as well as final tissue integration [37]. There were significantly higher numbers of adherent cells on the peek samples with a microporous surface after 72 h of culture compared to those on a smooth peek surface. However, good adherence on a rough surface does not necessary guarantee a perfect mechanical combination of the implant with the soft tissue, as the ultimate clinical goal.

Therefore, besides the surface structure, the overall design of the peek implant is also an important consideration for the stable and long-term integration of an implant and soft tissue. Based on the in vitro results with HSFs, we conducted an in vivo experiment to verify our hypothesis that an internal 3D cross-linked structure of the peek implant would provide a suitable channel to promote the growth of soft tissue and thus facilitate stable mechanical combination. We chose the simple and relatively stable internal net structure as a cross-linked channel for soft tissue growth. In support of our hypothesis, the rabbits transplanted with a smooth peek showed a fibrous capsule between the implant and soft tissue, which was not formed in the acid-etched peek groups and in the groups transplanted with peek implants of a cross-linked structure. Moreover, the separation test demonstrated poor mechanical combination between the soft tissue and peek implants without an internal cross-linked structure even when the surface was acid-etched. The macroporous surface thus provided sufficient space for the growth of blood vessels to ensure an appropriate nutrition supply for the soft tissue to grow into the peek implant.

Given the importance of the internal 3D structure, it is essential to ensure that such a specimen can be readily produced. Three-dimensional printing can realize both stable and anomalous external and internal structures [38]. At present, it is not possible to use CAD/CAM technology alone to manufacture objects with a fine internal structure [39,40]. Although injection molding can be used to manufacture peek implants with a definite shape, substitute models with a fine structure can be made by technicians, but the fine internal structure cannot be reconstituted [41,42]. Therefore, in the present study, we combined 3D printing and injection-molding technologies to fabricate peek specimens with an internal cross-linked structure, which completely reconstituted the structure designed with computer software.

The peek specimens with a microporous surface, and cross-linked internal structure improved the functionalities of HSFs and enhanced the mechanical combination with soft tissue in vivo, demonstrating good potential for clinical application of peek implants. Nonetheless, more studies, including animal experiments, are needed to confirm the optimal shape of the internal 3D structure, and the influence of various shapes on the functionalities of the epithelium and mechanical combination with a soft tissue to ensure an appropriate biological seal.

## 4. Materials and Methods

### 4.1. Sample Preparation

Medical-grade PEEK (Ketron LSG, Quadrant EPP, Baltimore, MA, USA) materials were used in this study. The peek samples were prepared with a cross-linked structure and a microporous surface manufactured by 3D printing and acid-etching, respectively. The cross-linked structure was first designed with Materialize Magic 22.0 software, and then the designed STL file was printed with a 3D printer (Eden 260, Stratasys, Merrimack, NH, USA) to produce the resin substitute model. The printed resin was then used to replace the wax model, and the peek specimen with a cross-linked structure was obtained through injection-molding (for2press, Bredent, Munich, Germany). Firstly, the resin model was fixed on the casting ring, and then the embedding material was mixed with the following proportion: 200 g embedded powder (for2press, bredent, Munich, Germany), 29 mL Embedded fluid(for2press, bredent, Muncih, Germany), and 24 mL water, this process lasted 1.5 min, then the embedding material was poured into casting circles until the casting ring was full. Then let it stayed at room temperature for 30 min, and then put the casting ring into the muffle furnace (Renfert, Magma, Munich, Germany). The furnace temperature raised up to 850 °C, and then kept 40 min at 850 °C, then the furnace temperature dropped to 400 °C and kept for 1 h. One hour later, peek raw material 1 g more than the total mass of resin models was added into the casting channel of the embedded molds, and was kept in a muffle furnace at 400 °C for 15 min. Then the embedded mold was taken out and placed on the injection molding machine and injection was started. The injection was finished after vacuum injecting for 3 min and kept at room temperature and pressure for 40 min. The embedded mold was rinsed with running water and the embedded material was removed. The injection-molded peek specimens were then cleaned with sand-blasting, and then, all the samples were washed with deionized water for 2 h in an ultrasonic oscillator (B3500S-MT, Branson, Danbury, CT, USA).

The peek specimens used for in vitro testing were 15 × 2-mm-thick disks designed to press-fit into the bottom of 24-well tissue culture plates. According to the macropore diameters, the peek specimens were divided into four groups: non-pore group (P), the pore diameter of 1 mm (P1.0), the pore diameter of 1.5 mm (P1.5), and pore diameter of 2.0 mm (P2.0). The peek specimens were gradually polished after removing residual embedded materials until the surface was as smooth as a mirror. The four groups of peek specimens were then etched in 98% concentrated sulfuric acid for 5 min each to obtain groups with microporous surfaces SP, SP1.0, SP1.5, and SP2.0, respectively. After etching, the peek specimens were cleaned in sequence with deionized water, acetone, and anhydrous ethanol to remove residual concentrated sulfuric acid from the surface. To ensure the maximum removal of concentrated sulfuric acid from the surface of the specimens, they were then placed in a 120 °C ventilated drying oven for 4 h. Finally, the specimens with and without acid etching were thoroughly cleaned and stored after high-temperature (121 °C) and high-pressure (0.12 MPa) sterilization for subsequent tests.

### 4.2. Surface Characterization

The surfaces of the prepared peek specimens were first observed by scanning electron microscopy (SEM; S4800, Hitachi, Tokyo, Japan). The peek specimens without a cross-linked structure, groups P and SP, were used to examine the difference between a rough surface with micropores after acid etching and a smooth surface without micropores. After dehydration by a gradient of 30%, 50%, 70%, 80%, and 90% anhydrous ethanol, the specimens were dried and sprayed with gold, and finally observed under SEM and energy dispersive spectrometry (EDS; X-Max, Horiba, Kyoto, Japan). The specimens were also subjected to atomic force microscopy (AFM, Vecco Instrument, Totonto, ON, Canada) in tapping mode with a probe (AR5-NCL, NANOSENSOR, Neuchâtel, Switzerland,) to compare the 3D morphology before and after acid etching. The hydrophilicity of the peek specimens was tested by measuring the contact angle with a contact angle goniometer (EASY DROP K100, KRUSS Co., Hamburg, Germany) between deionized water and the specimen’s surface.

### 4.3. Mechanical Properties

The compressive strength of the eight peek specimens (P, P1.0, P1.5, P2.0, SP, SP1.0, SP1.5, and SP2.0) was determined with an electronic universal testing machine (AGS-10kNG, Jindao, China); *n* = 6. First, we placed the specimen on the test board, and then pressed down at a rate of 3 mm/min until it cracked.

### 4.4. Combination of the Peek Implant with Soft Tissue In Vitro

#### 4.4.1. Culture of HSFs

Primary HSF cells were isolated and cultured, as previously described [43]. The HSFs were cultured in Dulbecco’s modified Eagle medium (DMEM, HyClone, Logan, UT, USA) with 10% fetal bovine serum (HyClone, Logan, UT, USA) and 1% penicillin/streptomycin (Sigma, St. Louis, MO, USA) at 37 °C in a humidified condition with 5% CO_2_ and 95% air. The cells were used at passages 3–8. For different assays, the cells were seeded onto the experimental substrates and placed in the 48-well polystyrene cell culture plate at a density of 3500 cells/cm^2^ for the cell adhesion assay and at a density of 1000 cells/cm^2^ for the other assays. The cell culture media were replaced every two days.

#### 4.4.2. Cell Adhesion Assay

Peek specimens from the P and SP groups were placed in 24-well plates, and then the HSFs were spread onto the surface of the specimens at a density of 1 × 10^4^ cells/well. High-glucose DMEM (HyClone, Logan, UT, USA) with 10% fetal bovine serum (FBS, HyClone, Logan, UT, USA) and 1% penicillin/streptomycin (Sigma, St. Louis, MO, USA) was added to the wells and placed in incubators with 5% CO_2_ at 37 °C. After 3 days, the culture medium was aspirated, the cells were washed with phosphate-buffered saline (PBS) three times, and the specimens were fixed with 2.5% glutaraldehyde. After 24 h, the glutaraldehyde was absorbed, the cells were re-washed with PBS three times. After dehydrating with an alcohol gradient, the specimens were dried, gold-sprayed, and observed with SEM.

#### 4.4.3. Cytotoxicity Assay

The cell proliferation was used as an index of cytotoxicity in the culture media. Peek specimens without and after acid corrosion were immersed in the cell culture medium, and then the culture medium soaked with peek specimen was used to culture HSF cells and observe the cell proliferation. The concentration of cells seeded onto the specimen substrate was cells cm −2 per substrate for cell in a standard 24-well culture plate under standard cell conditions. Cell viability was assayed with a Cell Counting Kit-8 (Dojindo Molecular Technologies Inc, Kumamoto, Japan). Briefly, after 1, 2, 3, 4, 5, 6, 7, and 8 days of culture, the culture medium was moved, and 200 mL of fresh culture medium mixed with 20 µL of Cell Counting Kit-8 reagent was added into each well. Then, the samples were incubated for 2 h, according to the manufacturer’s instructions. Besides, the same volume of culture medium and Cell Counting Kit-8 reagent without cells was also incubated as the background control. An aliquot (150 mL) of the incubated medium was pipetted into a 96-well plate, and the absorbance at 450 nm was measured for each well as above. All experiments were performed with a sample size of *n* = 6.

#### 4.4.4. Extracellular Matrix-Related Gene Expression

After the specimens of the P and SP groups were placed in the 24-well plate, the HSFs were added to the wells as described above, and then the expression of extracellular matrix-related genes was evaluated using real-time polymerase chain reaction (PCR).

The cells were seeded onto the experimental substrates in 24-well plates at a density of 1 × 10^4^ cells/well, cultured for 5 days, and then harvested using TRIzol reagent (Life Technologies Corporation, Carlsbad, NY, USA) to extract RNA. One microgram of RNA from each sample was reverse-transcribed into complementary DNA using the Prime Script^TM^ RT reagent kit (Takara Bio Inc., Otsu, Japan) using forward/reverse primers for the selected genes (Table 1). The real-time PCR analysis of genes was performed on the Bio-Rad iQTM5 multicolor real-time PCR Detection System using SYBR R Premix Ex Taq^TM^ II. The data were analyzed using the iQTM5 Optical System Software Version 2.0 (Bio-Rad, Carlsbad, CA, USA). The relative expression levels of target genes were normalized to the expression of the housekeeping gene *GAPDH*.

### 4.5. Combination of the Peek Implant with Soft Tissue in Vivo

All experimental protocols involving animal cells, animal experiments, and human cells were approved by the Ethics Committee of the Fourth Military Medical University, and the approval number is kq-019. To further test the ability of peek with a cross-linked structure and microporous surface to combine with soft tissue, we used transplant experiments in animals implanted with peeks of groups P, P1.0, P1.5, P2.0, SP, SP1.0, SP1.5, and SP2.0.

Ten male New Zealand white rabbits (weighing 3 kg each) were purchased from the animal center of the Air Force Medical University and fed in a clean environment for one week before the experiment. The rabbits were anesthetized with pentobarbital sodium (0.3 mL/kg), and the hair on the back and legs was removed and cleaned. The rabbits were disinfected with iodide and spread on sterile towels. A 20-mm cut was made in the back skin of the rabbit, and the subcutaneous tissue and skin were separated to form a cystic cavity. The prepared peek specimens were placed into the cystic cavity and sutured. Each rabbit was transplanted with 8 specimens in total, which came from 8 different groups. The animals were sacrificed at 4 weeks after transplantation, and samples (10 of 5 in each group) taken from the rabbits were fixed in a 4% paraformaldehyde solution. After 48 h, the samples were rinsed with tap water for 4 h, and then dehydrated for 7 days. Next, plastic dipping the samples was performed in a 45 °C water bath for 24 h and the embedded samples were cut into wafers of 300 µm thickness, with a diamond wire saw (EXAKT 312, Munich, Germany). Then, hematoxylin and eosin (HE) staining was performed on the hard tissue sections of the samples to evaluate whether the peek specimen and soft tissue had combined. The rest of the 5 samples of each group were used to perform the separation test. We used the single-column electronic universal testing machine (EZ-SX, Daojin, Tokyo, Japan) to test the force when the specimen and the soft tissue were separated. First, the sample was fixed at the lower end of the test machine and the upper end of the clamping part clamps the soft tissue part, and then the separation test procedure was performed until the soft tissue combined with the specimen was separated. 

### 4.6. Statistical Analysis

The comparisons among the eight groups were analyzed using a one-way analysis of variance (ANOVA, SPSS20.0 version, Chicago, IL, USA) and Tukey post-hoc analysis for pairwise comparisons (95% confidence interval). All data are expressed as mean ± standard error (SE). *p* < 0.05 was considered to indicate statistical significance.

## 5. Conclusions

We have provided the first demonstration that a peek implant with an acid-etched microporous surface promoted the early integration with soft tissue. The internal cross-linked structure fabricated by 3D printing and injection-molding technologies was conducive to the formation of right mechanical combination between the peek implant and the surrounding soft tissue, which effectively prevented necrosis of the soft tissue growing into the implant. Based on the results of compressive resistance in vitro and animal experiments, the peek specimen of group SP1.5, with a microporous surface, and internal cross-linked structure appears to be the most suitable design, offering a new direction for the application of peek in surgical repair.

## Figures and Tables

**Table 1:** Sequences of primers for RT-PCR.

Gene Name	Sequences of Primers
Type I collagen	Forward:5′-TCTAGACATGTTCAGCTTTGTGGAC-3′ Reverse: 5′-TCTGTACGCAGGTGATTGGTG-3′
Type III collagen	Forward:5′-GCAAATTCACCTACACAGTTCTGGA-3′ Reverse: 5′-CTTGATCAGGACCACCAATGTCATA-3′
VEGF	Forward:5′-GAGCCTTGCCTTGCTGCTCTAC-3′ Reverse: 5′-CACCAGGGTCTCGATTGGATG-3′
Integrin β	Forward: 5′-TGTGTCAGACCTGCCTTGGTG-3′ Reverse: 5′-AGGAACATTCCTGTGTGCATGTG-3′
Fibronectin	Forward:5′-ACCTACGGATGACTCGTGCTTTGA-3′ Reverse:5′-CAAAGCCTAAGCACTGGCACAACA-3′
ICAM-1	Forward:5′-TGAGCAATGTGCAAGAAGATAGC-3 Reverse: 5′-CCCGTTCTGGAGTCCAGTACA-3
GAPDH(house-keeping gene)	Forward:5′-GCACCGTCAAGGCTGAGAAC-3′ Reverse:5′-TGGTGAAGACGCCAGTGGA-3′
